# An Intervention to Reduce Bicycle Injuries among Middle School Students in Rural China

**DOI:** 10.3390/ijerph14070690

**Published:** 2017-06-26

**Authors:** Yanhu Ji, Yanru Ye, Yaogui Lu, Liping Li, Yang Gao

**Affiliations:** 1 Injury Prevention Research Center, Medical College of Shantou University, 22 Xin Ling Road, Shantou 515041, China; xiaohu123596@163.com (Y.J.); yyryezi@live.cn (Y.Y.); laoyaoguai6@163.com (Y.L.);; 2 Joint Shantou International Eye Center of Shantou University and the Chinese University of Hong Kong, Shantou 515041, China; 3 Department of Physical Education, Hong Kong Baptist University, Hong Kong, China; gaoyang@hkbu.edu.hk

**Keywords:** rural area, middle school students, bicycle injuries, intervention

## Abstract

This study aimed to evaluate the effectiveness of an intervention to reduce bicycle injuries among rural middle school students in China. A one-year cluster-randomized controlled trial was conducted with seventh grade students from six middle schools in two towns in rural Chaoshan, China. The two towns were randomly assigned to either the intervention or control group. Road safety education materials, two lectures on road safety, and a series of health education activities were delivered to 1312 students in the intervention group over one year, and the content of the intervention included traffic safety knowledge, methods of preventing bicycle injury and management of bicycle injuries. Questionnaires weere administered to the two groups before and after the intervention to measure the incidence, cognitions, and behaviors related to bicycle injuries. The pre-intervention incidence of bicycle injuries exhibited no significant difference between the two groups, while the difference reached significance after the intervention (χ^2^ = 13.409, *p* < 0.001). In the intervention group, the incidence decreased significantly after the intervention (χ^2^ = 8.137, *p* = 0.004), while no significant change was observed in the control group. Publicity and education intervention measures have certain short-term effects on the prevention of bicycle injuries among rural middle school students; we should approach intervention measures according to the characteristics of traffic injuries in different areas.

## 1. Introduction

Bicycling is a popular means of recreation, exercise, and transportation for children and youth worldwide. There are more than 800 million bicycles in the world, twice the number of motor vehicles [1]. One nationally representative study found that bicyclists had 2.3 times as many as fatalities and 1.8 times as many nonfatal injuries as motor vehicle occupants per 100 million person-trips [2]. For every two million trips, 600 injuries and one crash fatality are estimated to occur [3]. Bicycles are one of the main transportation modes in China’s vast rural areas and are mostly used by students as a means of transportation to school [4]. Of bicycle-related injuries, 20% requiring hospitalization involve motor vehicle collisions, but such collisions represent over 90% of all fatal cycling-related injuries [5]; therefore, bicycle riders represent a key group of traffic victims.

One of the main strategies used to prevent bicycle-related injuries is the promotion of helmet use, which can reduce the severity of bicycle-related injuries [6,7,8,9,10,11]. However, due to economic restrictions, many families in rural China cannot afford to buy these helmets. Another strategy of preventing bicycle-related injuries is environmental modification. A recent systematic review reported that purpose-built bicycle-specific lanes reduced crashes and injuries among cyclists [12]. Unlike urban environments, rural regions commonly have dirt roads and cement roads due to the backward economy and other factors, and motor vehicles, bicycles, and pedestrians share the available lanes. Pedestrian crossings are rare, and there are neither barrier-protected cycle tracks nor bike-designated crossing areas in rural China. Implementation of these preventive measures requires the participation of the government.

Bicycle safety education is an important method of bicycle injury prevention. Richmond SA [13] found that although education interventions may increase knowledge of cycling safety, this knowledge does not seem to translate into a decrease in injury rate or an improvement in bicycle handling ability and attitudes. This study was a systematic review of training interventions in the USA, UK, Australia, Canada, The Netherlands, and Sweden and the age range was 19 and younger. Even J. B. Carlin [14] found that educational intervention does not reduce the risk of bicycle injury in children and may in fact produce harmful effects for some children, perhaps due to the inadvertent encouragement to take risks or to bike with inadequate supervision. This was a case-control study, which was conducted in Australia from 1993 to 1996 and involved children who were 9 to 14 years of age. However, the intervention measures of health education, institutionalized management, and strict enforcement have been shown to prevent and control the incidence of bicycle injuries among middle school students in Shanghai [15].

In China, the laws and regulations regarding road traffic in 2011 clearly state that children under 12 years are not allowed to ride a bike on the road because roads are used for various vehicles, including both motor and non-motor vehicles. It is very dangerous for children to bike on the road, as they are still both physically and psychologically immature and therefore extremely vulnerable to traffic injuries. Research has shown that schools strictly comply with the state provisions. However, children under 12 years can ride a bike in their own neighborhoods with their parents or older siblings, as there are few motor vehicles in those areas.

In the suburbs and rural areas of Chaoshan region, bicycles are the main mode of transport among middle school students; 84.6% [4] of students ride a bicycle to school. However, the literature on bicycle injuries in this region is scarce. Whether an education intervention for bicycle injury is effective in Chaoshan, China, remains unknown. Therefore, we performed an epidemiological survey on bicycle injuries among middle school students and obtained the incidence and risk factors of these injuries in this region. To identify an effective intervention to reduce the occurrence of bicycle injury and to provide a scientific foundation for the government to establish injury prevention strategies, we chose two different towns according to their high and low rates of bicycle injury; in the intervention group (Fuyang town schools), we implemented the targeted intervention measures. In addition, we evaluated the intervention effects.

## 2. Methods

### 2.1. Study Population

Considering the purpose of this study and the actual situation in the region, we selected seventh grade students from three schools in Fuyang Town, Chaozhou, as the intervention group; implementation of traffic safety education was considered the main intervention measure. In addition, seventh grade students from three schools in Liangying Town, Shantou, were included as the control group. This study was approved by the Ethics Committee of the Medical College of Shantou University.

### 2.2. The Main Rationale Behind School Choice 

Chaozhou city is adjacent to Shantou city. The two cities are similar in terms of historical and cultural background, geographical environment, and climate characteristics as well as economic level. Liangying town in Shantou has an area of 72.4 square kilometers and is called the “Knitting town of China”, as many people there work in the textile industry. In 2010, the town had a population of nearly 200 thousand people. There are seven junior middle schools with approximately 3600 students. In 2009, the GDP of Liangying was 25.34 billion RMB, and the per capita annual net income was 4002 yuan. Fuyang town in Chaozhou has a developed private economy. In 2009, the town had a population of approximately 100 thousand people. Additionally, there are four junior middle schools, with approximately 2200 students. In 2002, the per capita annual net income was 4002 yuan. The total number of middle school students in the region was large enough to ensure the implementation of the intervention measures.

The schools were chosen to be relatively scattered according to location to prevent the influence of intervention activities on the surrounding schools. Through an early-stage epidemiology survey on bicycle injuries, we found that the incidence of bicycle injuries was higher among students in seventh grade than among those in other grades (Fuyang, 12.88%; Liangying, 15.67%). To better evaluate the efficacy of the intervention, we chose all seventh grade students from the six schools in the two towns as our research subjects. The control group did not receive any of the intervention measures. In the intervention group, traffic safety education was the main intervention measure used in the evaluation of the intervention effect.

### 2.3. Intervention Methods

#### 2.3.1. Two One-Hour Lectures (at Half-Year Intervals)

The lectures provided were mainly about traffic safety knowledge, injury prevention and how to address injuries. The lectures involved slides, classroom questions, animation, video and other forms, and each lecture lasted approximately one hour. Experienced university professors were available onsite to provide guidance, and the main lecturers were university-level scientific research personnel. The students’ parents were not involved in the intervention lecture.

#### 2.3.2. Distribution of Brochures

A brochure primarily on safety issues related to riding a bicycle and recognizing traffic signs was provided to the students. All information was based on a large body of evidence and was combined with the status quo of the local rural areas.

#### 2.3.3. Various Forms of Health Education Activities

We organized various forms of health education activities, including traffic safety bulletin competitions (once a month and for an hour each time), themed class meetings related to bicycle injuries (once a week and for an hour each time) and relevant knowledge contests (once a week and for an hour each time).

### 2.4. Intervention Evaluation

Before and after the intervention, we collected data through a questionnaire survey. The content of the bicycle injury questionnaire included the following: students’ general information (name, sex, age, family address, census register, nationality); basic facts regarding the bicycle injury (time injury occurred, time doctor was consulted, cause of the bicycle injury, description of the bicycle accident, site of the accident, and activities surrounding the bicycle injury); parents’ education level and employment; family members’ living situation (left-behind status); investigators; and the investigation date. In addition, the cognitive questionnaire included two main aspects: (1) knowledge and attitudes regarding traffic accidents, e.g., knowledge of the regulation preventing children under 12 years old from riding a bike, desire to study traffic accident prevention, and understanding of relevant traffic signs; and (2) perceptions related to bicycle riding, including use of crosswalks, management of bicycle breakdowns and focus on preventing traffic accidents. The validity and reliability of the questionnaire were analyzed, and the test-retest reliability and validity coefficients were both above 0.70.

### 2.5. Statistical Analysis

We input codes from the entire questionnaire into the entry database. All statistical analyses were performed using SPSS software version 19.0 (SPSS Inc., Chicago, IL, USA). Descriptive analysis was used to describe the students’ basic situation before and after the intervention. Differences between the intervention and control groups regarding the incidence of bicycle injury and the knowledge, attitudes, and perceptions (KAPs) of students toward safe biking before and after the intervention were compared using Chi-square tests. *P*-values less than 0.05 were considered statistically significant.

## 3. Results

### 3.1. Observations

Before the intervention, there were 587 male students and 725 female students in the intervention group and 566 male students and 476 female students in the control group. After the intervention, the intervention group included 492 male and 557 female students and the control group consisted of 345 male and 507 female students. There were no sex differences in the response or follow-up rates (*p* > 0.05). The average age was 13.01 ± 1.2 years in the intervention group and 13.1 ± 0.8 years in the control group. There were no age differences between the intervention group and the control group (*p* > 0.05).

Before the intervention, there was no significant difference in the incidence of bicycle injuries between the intervention group and the control group, while this difference was significant after the intervention (χ^2^ = 13.41, *p* < 0.001). In the intervention group, the bicycle injury incidence decreased significantly after the intervention (χ^2^ = 8.137, *p* = 0.004), while no significant change was found in the control group (Table 1).

### 3.2. Biking Safety in the Intervention Group

There was a significant difference in traffic safety knowledge before and after the intervention in the intervention group. Specifically, 64.1% of students knew that “children under 12 years old are not allowed to ride a bicycle”; the number of students who wanted to learn traffic accident prevention increased from 86.3% to 89.4%; and the number of students who knew traffic signs was higher after the intervention (*p* < 0.01). After the intervention, 90.1% of students wanted to study traffic accidents; 80.2% of students began to pay attention to how to prevent traffic accidents; and the number of students who said that they “walk on the right side of the road” increased from 92.5% to 95.4%. The number of students who did not use crosswalks was significantly lower than the number pre-intervention (*p* < 0.05); however, there was no significant difference in students’ knowledge of what to do if their bicycle broke down (*p* > 0.05) (Table 2).

### 3.3. Biking Safety in the Control Group

There were no significant differences in behavior among students in the control group. However, in terms of changes in knowledge, the number of students who knew that children under 12 years old were not allowed to ride a bicycle increased from 35.6% to 41.5%. The number of students who wanted to learn about traffic accidents increased (*p* < 0.01). However, there were no significant differences in the other variables (*p* > 0.05) (Table 3).

## 4. Discussion

There were no age differences between the intervention group and control group (*p* > 0.05), potentially because we selected only seventh grade students as the research subjects; more schools and grades should be included in future studies. The difference between the intervention group and control group in the incidence of bicycle injuries reached significance after the intervention (χ^2^ = 13.41, *p* < 0.001). The intervention played an important role in reducing the incidence of bicycle injuries among students in this study.

In Table 2, we can see that there was a significant difference in knowledge of traffic safety after the intervention in the intervention group. For example, 64.1% of students knew that “children under 12 years old were not allowed to ride a bicycle”. Roads are used by various trackless vehicles as well as pedestrians. Children under 12 years old can ride or learn to ride a bike with their parents or older siblings only in their own neighborhoods, where there are fewer motor vehicles. All the students in this study were older than 13 years old, and they were therefore allowed to ride a bike to school. Cycle tracks (physically separated paths exclusive to bicycles that are placed along roads) exist and continue to be built in the Netherlands, where 27% of all trips are made by bicycle [16]. However, the situation in the Netherlands is not consistent with that in rural China.

Attitudes toward learning about traffic injuries also changed in the intervention group students. For example, after the intervention, the number of students who began to pay attention to how to prevent traffic accidents increased from 73.5% to 80.2%. Regarding behaviors, although people are instructed to walk on the right side of the road facing oncoming traffic, many people still walk in a haphazard manner. After the intervention, 95.4% of students walked on the right side of the road, representing an increase in adherence to this behavior. The intervention measures had a very important effect on students’ awareness of road safety knowledge and attention to traffic injuries. However, the effect was limited in terms of changes in behavior. This limited impact may be related to the short research time. Nonetheless, the findings indicate that we should provide in-school education on emergencies to help students better protect themselves when traffic accidents occur.

Table 3 indicates that the effects of the intervention may have been related to the increase in students’ age or the broadening of their knowledge. The results may also suggest that the school made some effort to provide safety education for students. The number of students willing to learn how to prevent traffic accidents increased in the control group; this finding shows that although students were eager to learn the relevant knowledge, the schools may have been deficient in providing the relevant education. Injury intervention studies have become more widespread in China, and health education interventions have been effective in controlling injuries among Chinese primary and middle school students; therefore, these interventions are worth promoting [17,18,19,20,21,22]. One study developed health education and health promotion intervention activities to improve high school students’ ability to prevent and control traffic accidents and achieved good results [23]. Another study performed a variety of health education activities regarding road traffic laws for one year in intervention schools; this study improved students’ ability to prevent and control road traffic injuries [24]. 

In practice, implementing health education as the leading intervention factor and combining it with skill training and the removal of hidden dangers has been shown to effectively control the occurrence of student injuries. However, because of the shortage in research funding in developing countries, the uncertainty of the intervention effects, the enthusiasm of research staff, the prioritization and awareness of the topic in the government and other reasons, the effectiveness of many proven methods cannot be confirmed in developing countries. Researchers [25] have reported that promoting safety belt use is effective; however, it remains difficult to conclude whether other intervention measures have the same effect. Therefore, it is particularly important for countries to take different approaches to interventions.

Unlike in some foreign studies [13], in our intervention study, the education method was found to increase knowledge of cycling safety, resulting in a decrease in injury rate and a certain improvement in bicycle handling ability and attitudes. These results indicate that the education intervention method was effective in preventing bicycle injuries among rural middle school students in Chaoshan, China.

## 5. Limitations

Due to time limitations and funding conditions, this study selected only seventh grade students as the research subjects. Therefore, whether the results of the study can be applied to other high school students remains to be determined.

The instructional materials might have had only a short-term impact after the students listened to the two one-hour lectures and received the brochure. Further studies should be conducted to examine the long-term effects.

The two samples were not perfectly matched at baseline. For example, in the intervention group (Fuyang), 56.3% knew that 12-year-olds were not allowed to bike to school, while in the control group (Liangying), 35.6% knew that 12-year-olds were not allowed to bike to school at baseline. In the pre-intervention group, 71.6% knew the meaning of traffic signs, while in the pre-control group, only 39.6% knew these meanings. The large imbalances between the two groups may have threatened the results by underestimating the intervention’s effect through two potential mechanisms: (1) the higher level of knowledge in the intervention group at baseline may have led to a smaller improvement after the intervention and (2) the relatively lower injury rate in the pre-intervention group (at least partially due to its higher knowledge level) may have also resulted in a relatively lower decrease in the injury rate after the intervention. 

One limitation of the study was the relatively high withdrawal rates (20% and 18.2% in the intervention and control groups, respectively), which may have threatened the study results, as we do not know if the intervention would work well among dropouts. Students withdrawing from the study mainly did so because of the potential negative influences of study participation on their academic studies. Some other students dropped out because they thought that the intervention was not appealing. In addition, we failed to obtain full support from some of the study schools.

Despite these limitations, this study confirmed that an education intervention has certain effects on bicycle injuries among middle school students in rural China.

## 6. Conclusions

Bicyclist injuries have been found to be the leading type of all injuries using different traffic tools. The highest death rate in China was over eight times higher than that in the United States [9]. We chose two different towns as the intervention sites, and we implemented the targeted education intervention measures in the intervention group. We found that the incidence of bicycle injuries decreased significantly in the intervention group after the intervention. The knowledge, attitudes, and perceptions (KAPs) toward safe biking increased after the intervention among students in the intervention group. This study shows that publicity and education interventions have certain short-term effects on reducing bicycle injuries among rural middle school students; therefore, we should approach intervention measures according to the characteristics of traffic injuries in different areas.

## Figures and Tables

**Table 1:** A comparison of the bicycle injury rate in the intervention group and control group.

	Intervention Group (Fuyang Town School)		Control Group (Liangying Town School)		χ^2^	*p*
	Injury Incidence (%)	Number	Injury Incidence (%)	Number		
Before intervention	12.88	1312	15.67	1042	3.656	
After intervention	9.14	1049	14.54	852	13.41	*p* < 0.01

**Table 2:** The KAPs toward safe biking before and after the intervention among students in the intervention group.

Item	Before Intervention		After Intervention		*p*
	*N*	Rate (%)	*N*	Rate (%)	
**Use of crosswalks**					
Never	532	44.1	488	46.8	
Seldom	367	30.5	330	31.6	
Sometimes	180	14.9	120	11.5	*p* < 0.05
Often	64	5.3	35	3.4	*p* < 0.05
Always	62	5.1	30	2.9	*p* < 0.01
**Total**	1205	100	1043	100	
**How to manage bicycle if it breaks**					
Fix as soon as possible	920	75.6	1002	95.6	
Disregard, keeping it for use	296	24.4	46	4.4	
**Total**	1216	100	1048	100	
Walking in roads without sidewalks *					
Walk on the right side of the road	1126	92.5	1000	95.4	*p* < 0.01
Walk on any side	91	7.5	48	4.6	
**Total**	1217	100	1048	100	
Are you concerned about how to prevent traffic accidents *					
No	323	26.5	200	19.8	*p* < 0.01
Yes	897	73.5	810	80.2	
**Total**	1220	100	1010		
Do you know of traffic accident injuries *					
Yes	1050	87.4	924	90.1	*p* < 0.01
No	152	12.6	91	10.9	
**Total**	1202	100	1015	100	
Do you know that 12-year-olds are not allowed to ride a bicycle *					
Correct answer	682	56.3	668	64.1	*p* < 0.01
Wrong answer or did not know	530	44.7	374	35.9	
**Total**	1212	100	1042	100	
Do you want to learn traffic accident prevention *					
Yes	1015	86.3	930	89.4	*p* < 0.05
No	161	13.7	110	10.6	
**Total**	1176	100	1040	100	
Meaning of traffic signs *					
Correct answer	864	71.6	802	77.0	*p* < 0.01
Wrong answer	343	28.4	240	23.0	
**Total**	1207	100	1042	100	

Note: KAPs: Knowledge, Attitudes and Perceptions *: *p* < 0.05.

**Table 3:** The KAPs toward safe biking before and after the intervention among students in the control group.

Item	Before Intervention		After Intervention		*p*
	*N*	Rate (%)	*N*	Rate (%)	
**Use of crosswalks**					
Never	546	52.4	450	52.94	
Seldom	283	27.2	250	29.4	
Sometimes	137	13.1	95	11.52	
Often	35	3.4	30	3.53	
Always	41	3.9	22	2.59	
**Total**	1042	100	850	100	
**How to address bicycle if it breaks.**					
Fix as soon as possible	972	93.6	794	93.2	
Disregard, keeping for use	66	6.4	58	6.8	
**Total**	1038	100	852	100	
Walking on roads without sidewalks *					
Walk on the right side of road	958	96	822	97	
Walk on any side	40	4	25	3	
**Total**	998	100	847	100	
Are you concerned about preventing traffic accidents *					
No	326	31.3	259	30.6	
Yes	714	68.7	588	69.4	
**Total**	1040	100	847	100	
Do you know about traffic accident injuries *					
Yes	832	83.5	721	84.8	
No	165	16.5	129	15.2	
**Total**	997	100	850	100	
Do you know that 12-year-olds are not allowed to ride bicycles *					
Correct answer	363	35.6	350	41.5	*p* < 0.01
Wrong answer or did not know	657	64.4	495	58.5	
**Total**	1020	100	845	100	
Do you want to learn about traffic accident prevention *					
Yes	825	80.1	677	84.7	*p* < 0.05
No	206	19.9	122	15.3	
**Total**	1031	100	799	100	
Meaning of traffic signs *					
Correct answer	404	39.6	361	43.2	
Wrong answer	615	60.4	476	56.8	
**Total**	1019	100	837	100	

Note: KAP: Knowledge, Attitude and Perception *: *p* < 0.05.
